# Viral burden, inflammatory milieu and CD8^+^ T‐cell responses to influenza virus in a second‐generation thiazolide (RM‐5061) and oseltamivir combination therapy study

**DOI:** 10.1111/irv.12776

**Published:** 2020-06-25

**Authors:** Simone Nüssing, Edin Mifsud, Luca Hensen, Marios Koutsakos, Zhongfang Wang, Lukasz Kedzierski, Francesca Mercuri, Jean‐Francois Rossignol, Aeron C. Hurt, Katherine Kedzierska

**Affiliations:** ^1^ Department of Microbiology and Immunology Peter Doherty Institute for Infection and Immunity University of Melbourne Parkville Victoria Australia; ^2^ Victorian Infectious Diseases Reference Laboratory (VIDRL) Peter Doherty Institute for Infection and Immunity WHO Collaborating Centre for Reference and Research on Influenza Parkville Victoria Australia; ^3^ Romark Laboratories L.C. Tampa FL USA

**Keywords:** Influenza, nitazoxanide, oseltamivir, RM‐5061, thiazolide

## Abstract

**Background:**

Influenza viruses cause significant morbidity and mortality, especially in young children, elderly, pregnant women and individuals with co‐morbidities. Patients with severe influenza disease are typically treated with one neuraminidase inhibitor, oseltamivir or zanamivir. These antivirals need to be taken early to be most effective and often lead to the emergence of drug resistance and/or decreased drug susceptibility. Combining oseltamivir with another antiviral with an alternative mode of action has the potential to improve clinical effectiveness and reduce drug resistance.

**Methods:**

In this study, we utilized a host‐targeting molecule RM‐5061, a second‐generation thiazolide, in combination with oseltamivir to determine whether these compounds could reduce viral burden and understand their effects on the immune response to influenza virus infection in mice, compared with either monotherapy or placebo.

**Results:**

The combination of RM‐5061 and OST administered for 5 days after influenza infection reduced viral burden at day 5 post‐infection, when compared to placebo and RM‐5061 monotherapy, but was not significantly different from oseltamivir monotherapy. The inflammatory cytokine milieu was also reduced in animals which received a combination therapy when compared to RM‐5061 and placebo‐treated animals. Antiviral treatment in all groups led to a reduction in CD8^+^ T‐cell responses in the BAL when compared to placebo.

**Conclusions:**

To our knowledge, this is the first time a combination of a host‐targeting compound, RM‐5061, and neuraminidase inhibitor, OST, has been tested in vivo. This antiviral combination was safe in mice and led to reduced inflammatory responses following viral infection when compared to untreated animals.

## 1 BACKGROUND

Influenza viruses remain a global health burden, with influenza A (IAV) and B (IBV) strains co‐circulating and causing an excess 650 000 deaths annually.^1^, ^2^ Infants, the elderly, pregnant women, obese and indigenous populations are particularly at risk of severe disease outcomes. Antivirals are used to mitigate the disease and alleviate symptoms caused by influenza viruses. These compounds typically target various aspects of the viral replication cycle. Amantadine and rimantadine target the M2 ion channel of the influenza virus disrupting viral uncoating following entry into the host cell,^3^ while the neuraminidase inhibitors (NAIs), with oseltamivir (OST) being the most frequently prescribed, inhibit the release of newly formed virions.^4^ More recently a new influenza antiviral, baloxavir marboxil, which inhibits the polymerase acidic (PA) endonuclease, stopping cleavage of mRNA during the cap snatching process, has been licensed for use in otherwise healthy and high‐risk individuals in Japan, the United States and other countries.^5^ These antiviral drugs directly target the influenza virus, and therefore drug‐resistant viruses can emerge. This has been observed most dramatically with the widespread circulation of IAVs resistant to the adamantanes, and in 2008‐2009 the global circulation of oseltamivir‐resistant H1N1 viruses.^6^ Currently, the frequency of A/(H1N1)pdm09 viruses with reduced susceptibility to OST is between 1% and 3%.^7^, ^8^

An alternate approach, and one which is successful in HIV and malaria, is to use a combination of two or more drugs. Combination therapy can enable rapid viral clearance facilitating better clinical outcomes and reducing the likelihood of the emergence of viral resistance. These compounds can target host factors or viral proteins, as exemplified by the combination of NAIs and M2e ion channel blockers reducing viral burden by ~100% in vitro.^9^ In mice, a 1000‐fold reduction was observed in viral burden in animals treated with OST and rimantadine when compared to placebo‐treated animals.^10^

Previously, it has been shown that tizoxanide, the active metabolite of the anti‐parasitic drug nitazoxanide^11^, ^12^, ^13^(NTZ), was active against circulating influenza viruses, including those resistant to NAIs.^14^ Nitazoxanide, a first‐generation thiazolide, was licensed for use in the treatment of gut parasitic infections in the 1970s, but more recently has been repurposed to combat viral infections. Nitazoxanide can alter mitochondrial respiration^15^ and inhibit terminal hemagglutinin glycosylation in the endoplasmic reticulum thereby preventing viral exit.^16^

In the ferret model, we have shown that the combination of OST and NTZ reduced the incidence of infection and that if infected, virus remained in the upper respiratory tract and did not enter the lungs which is typically associated with severe disease {Mifsud, 2020 #19}. In this study, we investigated the effects of RM‐5061, a second‐generation thiazolide, developed to improve systemic absorption,^17^ in combination with OST on reducing viral burden and subsequent effects on the immune response to influenza virus infection.

We hypothesized that the combination of RM‐5061 + OST would have a greater impact on reducing viral burden and potentially altering immune responses to influenza virus infections via targeting different mechanisms of action in the host and viral replication cycle. Our results show that, the overall cytokine response was lower in animals undergoing RM‐5061 combination therapy when compared to glucose and RM‐5061 monotherapy but was comparable with OST‐treated animals. Antiviral treatment in all the groups led to a significant reduction in the influenza‐specific CD8^+^ T‐cell responses in the bronchioalveolar lavage fluid (BAL). Overall, in this study, we show that the combination of RM‐5061 + OST is as effective as OST monotherapy at reducing viral burden and inflammatory response in severe influenza disease.

## 2 METHODS

### 2.1 Mice

C57Bl/6 mice were bred and housed in the Biological Research Facility of the Peter Doherty Institute, Department of Microbiology and Immunology, the University of Melbourne under specific pathogen‐free conditions. Female mice used in experiments were between 6‐12 weeks of age and experiments were performed in approval by and accordance with the requirements of the University of Melbourne Animal Ethics Committee (1714106).

### 2.2 Viral infection and harvesting of tissues

Mice were anaesthetized with 2% of isoflurane in oxygen and infected intranasally with 1 x 10^4^ pfu of the laboratory‐adapted strain of IAV A/Hong Kong‐x31 (A/HKx31, H3N2) in 30 µL PBS. Mice were culled by CO_2_ asphyxiation on day 5 (d5) or day 7 (d7) post‐infection (pi) for immune analyses. BAL was performed by washing lungs of IAV‐infected mice with 1 mL HBSS. Lungs were perfused by cutting the pulmonary artery and injecting 5 mL of PBS into the right atrium of the heart before harvest. Single cell solutions from mLN and spleen were prepared by meshing organs through a 70 µm cell strainer. Lungs were processed with a tissue homogenizer, cell debris was spun down and supernatants were stored at −80°C until analysis for cytokine amounts and viral titres.

### 2.3 Drug treatment

Mice were treated twice daily via oral gavage with 3 mg of RM‐5061, 1 mg of Ost, or 3 mg of RM + 5061 + 1 mg of Ost in 180 µL of 50% Glucose or 50% Glucose only per dose.

### 2.4 Plaque assays

Plaque assays were performed in MDCK cell lines by seeding 1.2 x 10^6^ cells into 6‐well plates in 3 mL of MDCK medium and rested overnight at 37°C and 5% CO_2_. Lung homogenates from infected mice were diluted in 10x serial dilution and incubated with MDCK cell monolayers for 1 hour at 37°C for virus attachment. Agarose overlay medium was then added and plaques for plaque‐forming units (PFU) per lung were counted after 3 days.

### 2.5 Flow cytometry

Single cell suspensions from BAL, mLN and spleens were blocked with 2.4G2 supernatant and stained with different antibody mixes of anti‐CD3 (PerCPCy5.5; AlexaFluor 700), I‐Ab (Pacific Blue), CD45.2 (BV711, CD64 (PE), CD11b (BV605), Ly6G (PE‐Cy7), SiglecF (PECF‐594), CD11c (FITC), Ly6C (AlexaFluor 700), live/dead Aqua, TCR γδ (BV421), CD44 (APC‐Cy7), NK1.1 (PE), F4/80 (FITC, CD19 (FITC), and CD8 (PerCP‐Cy5.5). For D^b^NP_366_ and D^b^PA_224_ specific CD8^+^ T cells, samples were stained with PE‐ or APC‐conjugated H‐2D^b^ tetramers prior to antibody staining.

### 2.6 Cytokine bead array

Cytokine concentrations in supernatants from lung homogenates were quantified using the mouse BD Bioscience cytometric bead array following the manufacturer's instructions. Data were acquired using FACSCantoII and analysed using the flowjo software vs10 (Tree Star) and Prism 7 (graphpad software).

## 3 RESULTS

### 3.1 OST monotherapy and OST + RM‐5061 combination reduced body weight loss in mice following A/HKx31 influenza virus infection

**Figure 1:**
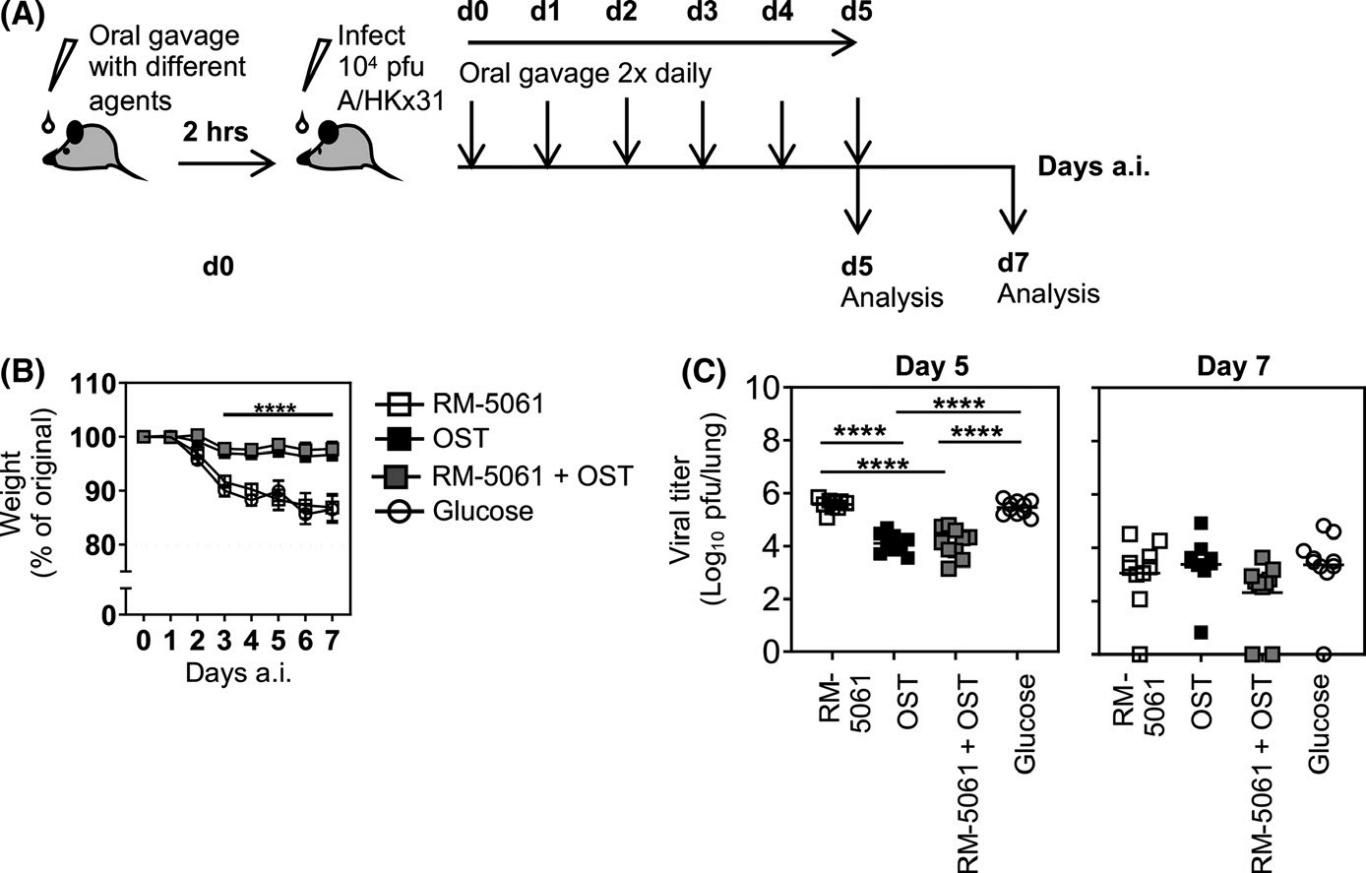
Reduced body weight loss and d5 viral load in mice on RM‐5061 + OST and OST therapy following A/HKx31 influenza virus infection. (A) Mice were treated twice daily with either RM‐5061 alone, OST alone, RM‐5061 + OST or placebo only for the course of 5 d after A/HKx31 infection with the initial dose administered 2 h prior to infection. (B) Body weight loss of mice across different treatment groups was monitored over the course of infection up to d7. (C) Viral titres of lung homogenates on d5 and d7 p.i. were quantified using the MDCK cell line plaque assay. Data are from two independent experiments with 5 mice per treatment group. One‐way ANOVA was performed between groups (****P ≤ .0001)

Mice were treated twice daily with either RM‐5061 (3.4 mg), OST (1 mg), RM‐5061 (3.4 mg) + OST (1 mg) or 50% Glucose only (placebo). The first dose was administered 2h prior to intranasal infection with 1 x 10^4^ pfu A/HKx31. Mice were monitored daily following viral infections, and animals were euthanized at d5 to d7 p.i.to determine viral burden (Figure 1A). Animals treated with either OST or RM‐5061 + OST maintained their overall body weight following infection when compared to the RM‐5061 and placebo groups (Figure 1B). Animals receiving RM‐5061 + OST and OST (mean viral titre 4.1 and 4.1 log_10_ pfu/lung, respectively) had significantly reduced viral titres in comparison to placebo and RM‐5061 monotherapy (mean viral titres 5.5 and 5.6 log_10_ PFU/lung, respectively; Figure 1C). By d7, no differences in viral titres were evident between all the treatment groups, suggesting an early effect of RM‐5061 + OST and OST either on viral clearance, or by directly suppressing viral replication when antiviral treatment was administered for up to d5 p.i..

### 3.2 Reduced NK cells and neutrophils in OST and RM‐5061 + OST‐treated animals

Given the importance of the innate immune system in controlling viral replication during the early stages of viral infection, we examined the levels of eosinophils (Siglec‐F^+^CD11c^‐^), neutrophils (Ly6G^+^CD11b^+^), DCs (I‐Ab^+^CD11c^+^), NK cells (CD3^‐^NK1.1^+^) and macrophages (CD64^+^) at the site of infection (BAL) and spleen at d5 and d7 pi.

**Figure 2:**
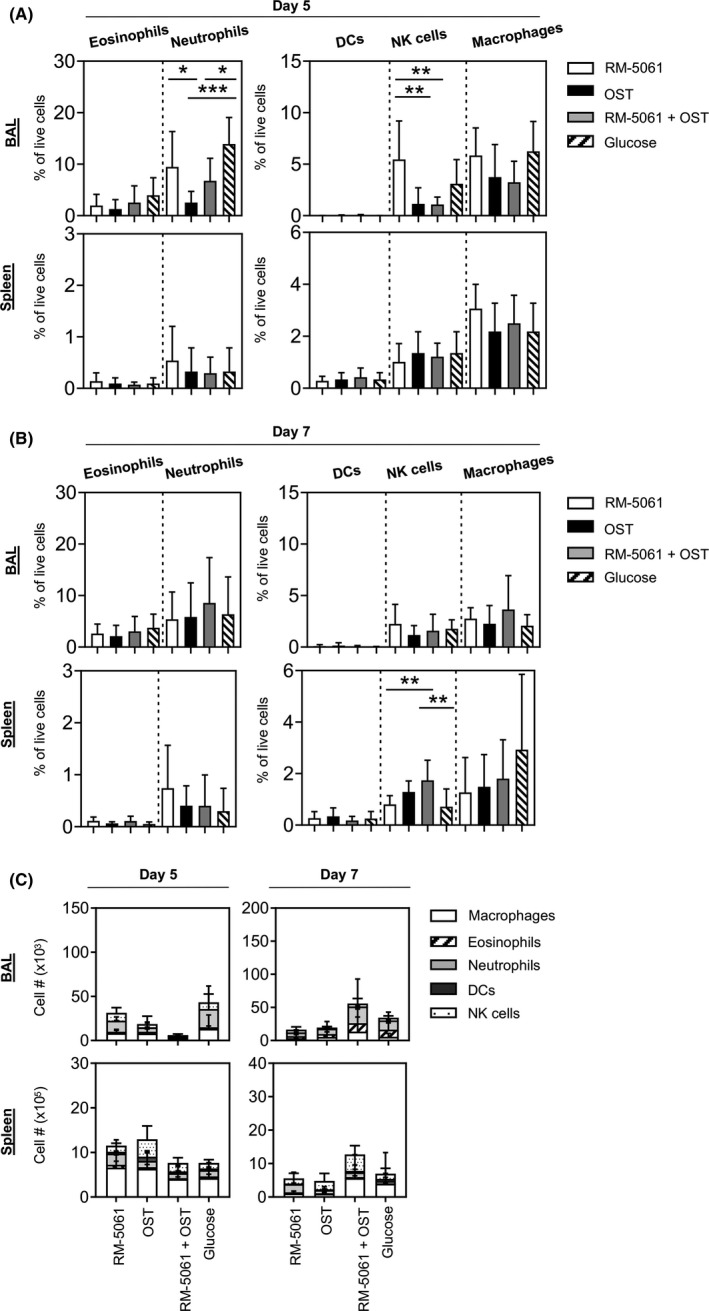
Reduced NK cells and neutrophils in RM‐5061 + OST‐treated and OST‐treated animals. Innate immune responses across different drug treatments and A/HKx31 infection were determined by staining for eosinophils, neutrophils, DCs, NK cells and macrophages in BAL and spleen. Frequencies at (A) d5 and (B) d7 as well as (C) total numbers of the cell subsets were graphed. Data are from two independent experiments with 5 mice per treatment group. One‐way ANOVA was performed between groups (*p ≤ .05; **P ≤ .01, ***P ≤ .001)

Despite observing a reduction in viral burden at d5 p.i., we found no differences in the levels of eosinophils, DCs and macrophages in the BAL (Figure 2A,C). However, we identified significantly lower frequencies of neutrophils in RM‐5061 + OST (mean 6.8%) and OST (mean 2.5%)‐treated mice compared with placebo (mean 13.9%) and/or RM‐5061 monotherapy (mean 9.5%) groups in BAL on d5 p.i. (Figure 2A) but not on d7 p.i. (Figure 2B). Although not significant, RM‐5061 monotherapy reduced the levels of neutrophils by 4.4% in comparison to placebo (Figure 2A). The percentages of NK cells were significantly reduced on d5 in RM‐5061 + OST‐treated mice (mean 1.1%) and OST monotherapy (mean 1.1%) groups compared with RM‐5061 monotherapy (mean 5.5%)‐treated mice (Figure 2A). There was an overall trend for animals treated with RM‐5061 + OST to have lower total numbers of innate immune cells in BAL and spleen on d5 p.i. when compared to other treatment groups (Figure 2C); however, this result was not statistically significant.

On d5 pi, we found significantly elevated levels of NK cells in spleens of the OST group (mean 3.9 x 10^5^) and on d7 pi for the RM‐5061 + OST group (mean 4.9 x 10^5^) when compared to placebo (d5, 1.2 x 10^5^; d7, 1.6 x 10^5^) and RM‐5061 (d5, 1.4 x 10^5^; d7, 1.5 x 10^5^)‐treated animals (Figure 2C). Trends in the different innate cell numbers between animals receiving either placebo or the combination RM‐5061 + OST were seen in the BAL on d5 p.i. (Figure 2C), suggesting a potential early effect of RM‐5061 + OST and OST therapies on innate immune responses during influenza infection.

### 3.3 Reduced RANTES in lungs of RM‐5061 + OST and OST‐treated mice during the course of influenza virus infection

**Figure 3:**
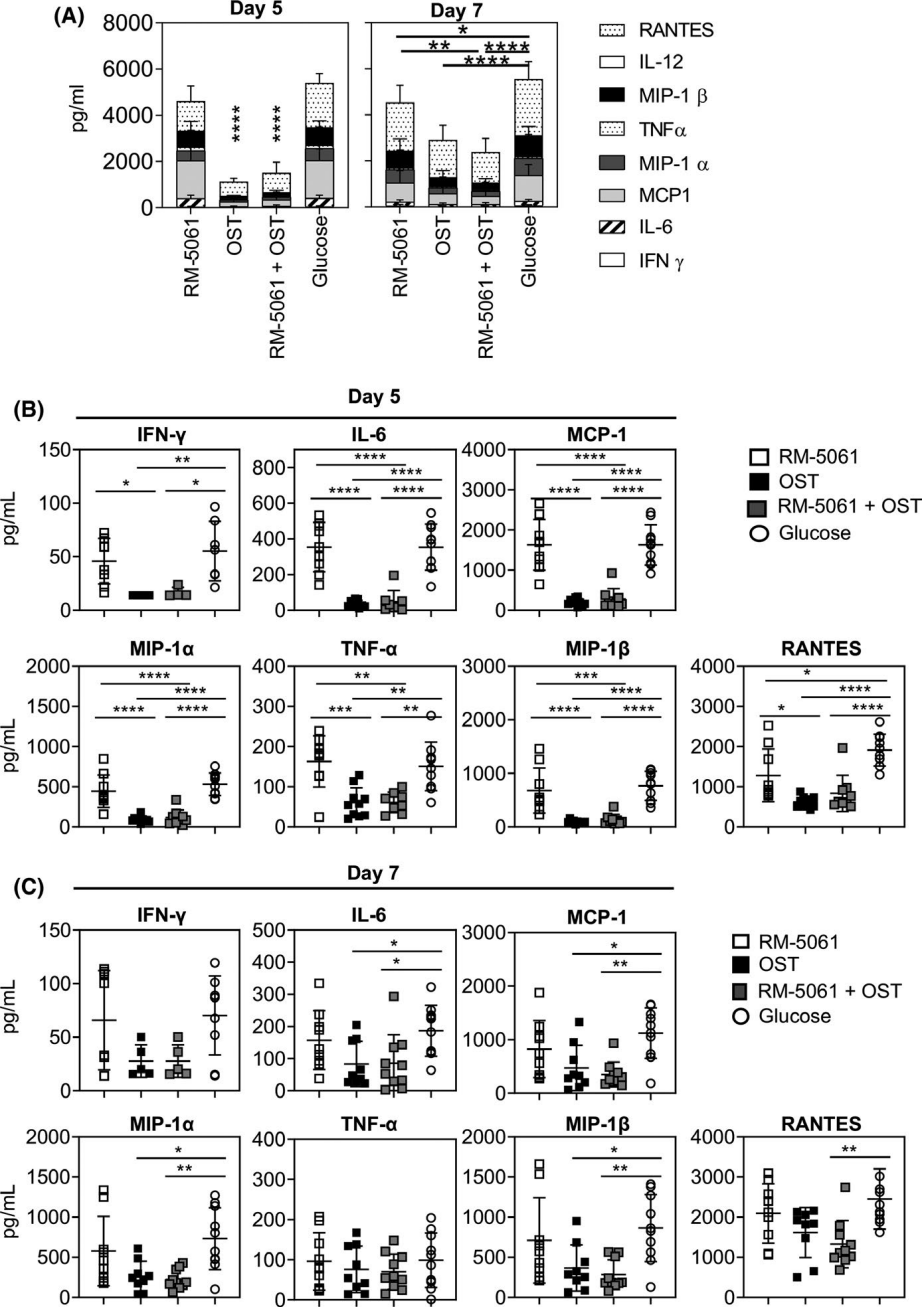
Reduced cytokine milieu in lungs of mice with RM‐5061 + OST and OST treatments. Cytokine levels in lungs of different drug treatment groups were assessed by CBA assays on lung homogenate supernatants on d5 and d7 after A/HKx31 infection with antibodies staining for IFN‐γ, IL‐6, MCP‐1, MIP‐1α, TNF‐α, MIP‐1β, IL‐12 and RANTES. (A) Combined cytokine/chemokine amounts are shown at d5 and d7 pi; Data for individual cytokine/chemokines are plotted on (B) d5 and (C) d7. Data are from two independent experiments with 5 mice per treatment group. One‐way ANOVA was performed between groups (*p ≤ .05; **P ≤ .01, ***P ≤ .001, ****P ≤ .0001). Undetectable amounts of cytokines were excluded from the analysis

Given the important role inflammatory cytokines and chemokines play in both influenza‐induced morbidity and mortality^18^ and recovery from influenza virus infection,^19^ we subsequently examined the levels of pro‐inflammatory cytokines RANTES, IL‐12, MIP1‐β, TNF‐α, MIP‐1α, MCP1, IL‐6 and IFN‐γ in the lungs using a cytokine beads array kit (CBA). Overall, the total amounts in pg/mL of cytokines present in the lungs of RM‐5061 + OST and OST‐treated mice were greatly reduced when compared to placebo and RM‐5061 monotherapy (Figure 3A). All the cytokines and chemokines were significantly reduced in RM‐5061 + OST and OST‐treated mice on d5 post‐infection (Figure 3B). Additionally, RANTES was also reduced in RM‐5061 monotherapy‐treated mice (mean 1283 pg/mL) compared to placebo animals (mean 1911 pg/mL) at this time point (Figure 3B). By d7 p.i., IL‐6, MCP‐1, MIP‐1α, MIP‐1β were significantly reduced in RM‐5061 + OST (mean IL‐6 86.2, MCP‐1 350.3, MIP‐1α 226.5, MIP‐1β 284.7 pg/mL) and OST monotherapy (mean IL‐6 83.6, MCP‐1 471.1, MIP‐1α 263.9, MIP‐1β 368.2 pg/mL) groups compared to placebo animals (mean IL‐6 186.8, MCP‐1 1121, MIP‐1α 733, MIP‐1β 864.1 pg/mL) but not when compared to RM‐5061 monotherapy (Figure 3C). In comparison with placebo animals (mean 2451 pg/mL), the levels of RANTES on d7 pi. were significantly reduced in RM‐5061 + OST‐treated mice (mean 1331 pg/mL). Overall, combination RM‐5061 + OST or OST monotherapy leads to a significant reduction in the amounts of cytokines and chemokines in the lung.

### 3.4 Reduced CD8^+^ T‐cell numbers in BAL of RM‐5061 and/or OST‐treated mice on d7 p.i

**Figure 4:**
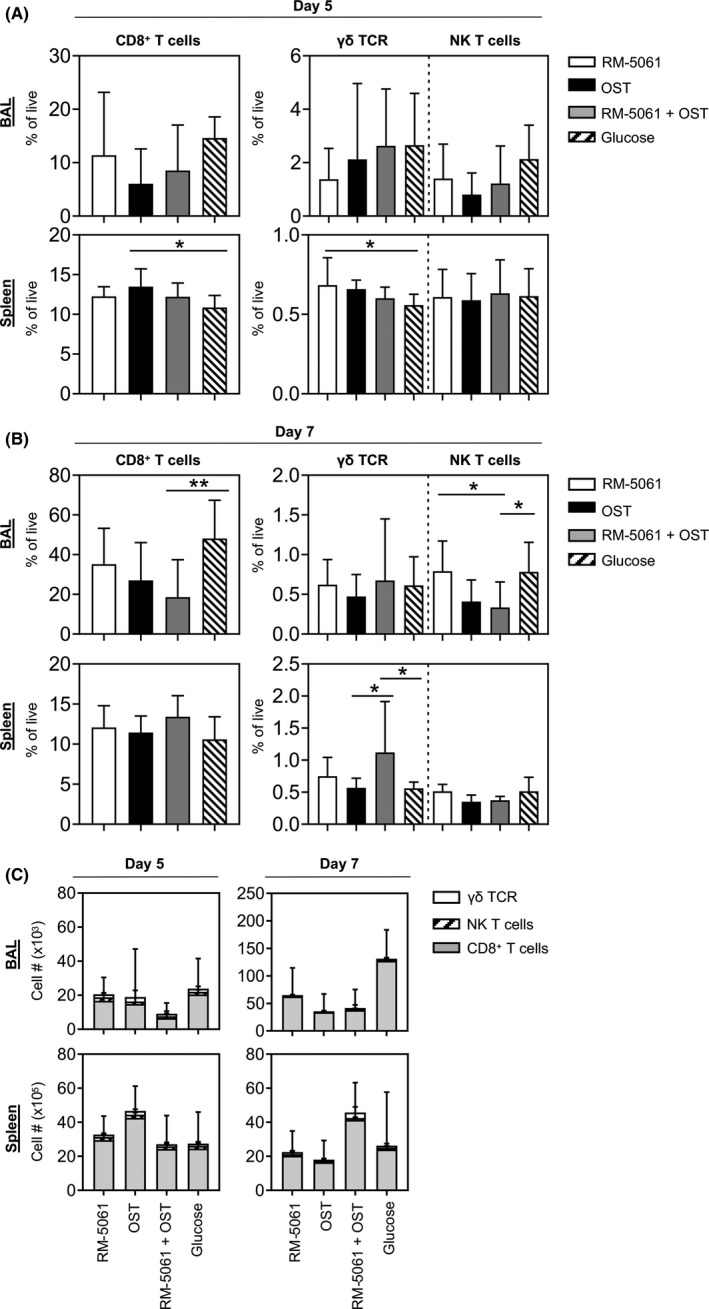
Adaptive T‐cell responses across different drug treatment groups after A/HKx31 infection. Mice across different drug treatment groups were assessed for (A, B) frequencies and (C) total numbers of CD8^+^ T cells, γδ, and NK T‐cell responses on (A, C) d5 and (B, C) d7 p.i. in BAL and spleen. Data are from two independent experiments with 5 mice per treatment group. One‐way ANOVA was performed between groups (*p ≤ .05)

The adaptive immune responses drive recovery from acute influenza virus infection and lead to the establishment of long‐lasting immune memory against subsequent viral encounters.^2^, ^20^, ^21^ This can be exemplified by CD8^+^ T cells, NKT and γδ T cells playing pivotal roles in combatting the disease caused by influenza virus infections and ultimately result in accelerated recovery times.^19^, ^20^, ^22^ As innate immune responses and viral load can affect the recruitment of adaptive immune cells, we investigated the numbers of CD8^+^ T, NKT‐ and γδ T cells in the BAL and spleen at d5 and d7 p.i.. No significant differences were found in the BAL 5 days after infection across any of the groups. In the spleen, however, animals treated with OST displayed higher frequencies of total CD8^+^ T cells (13.5%) when compared to placebo (10.9%) and RM‐5061‐treated animals had significantly greater frequencies of γδ T cells (0.7%) when compared to placebo (0.5%; Figure 4A).

There was a trend for RM‐5061 + OST‐treated animals to have lower percentages (Figure 4B) and total number of cells (Figure 4C) in the BAL on d5 p.i. compared with placebo. A significant difference was observed in the combination group on d7 p.i. (Figure 4B,C), with additionally RM‐5601 and OST alone showing significant lower CD8^+^ T‐cell numbers in the BAL on d7 p.i. (Figure 4C), whereas this effect was not present in the spleen on d7 pi. (Figure 4C). This late effect of OST or RM‐5061 + OST combination therapy was also seen for NKT cells, with reduced NK T‐cell percentages in the BAL of OST and RM‐5061 + OST groups on d7 p.i. (Figure 4B).

By d7, no differences in the draining mLN were detected in total CD8^+^ T, NKT and γδ T cells amongst all treatment groups (data not shown).

### 3.5 Antiviral therapies reduce influenza‐specific CD8^+^ T‐cell responses in the lung and spleen

**Figure 5:**
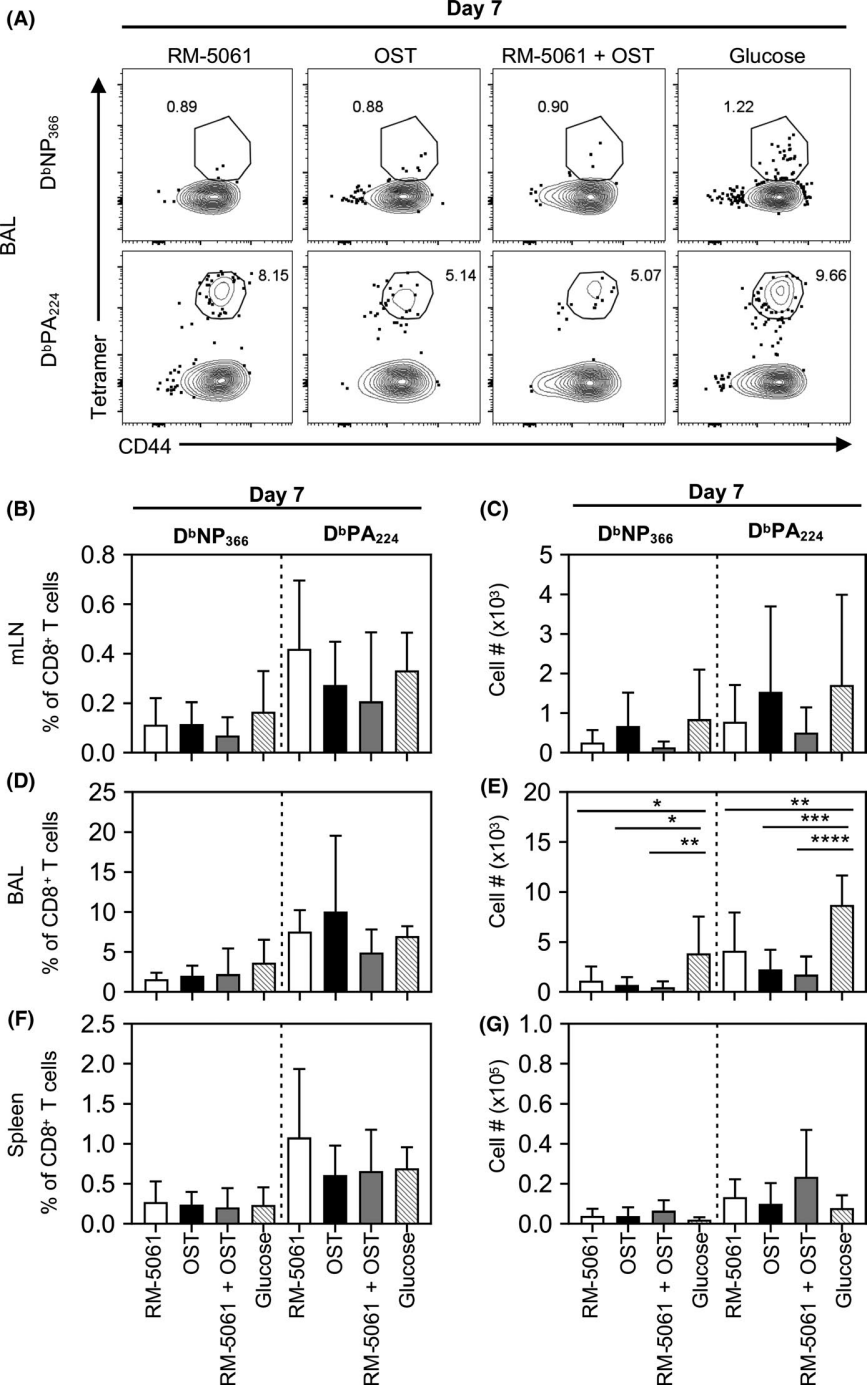
Diminished IAV‐specific T‐cell responses in all drug treatment groups after A/HKx31 infection. IAV‐specific immunodominant D^b^NP_366_^+^ or D^b^PA_224_^+^ T‐cell responses on d7 after A/HKx31 were determined as (A, B, D, F) frequencies and (C, E, G) total numbers by staining CD8^+^ T cells with the D^b^NP_366_ or D^b^PA_224_ tetramer in (B, C) mLN, (D, E) BAL and (F, G) spleen after administration of different drug treatments to the mice. Data are from two independent experiments with 5 mice per treatment group. One‐way ANOVA was performed between groups (*p ≤ .05; **P ≤ .01, ***P ≤ .001, ****P ≤ .0001)

Influenza‐specific CD8 T cells play an important role in recovery from influenza virus infection in mice and humans.^19^, ^23^, ^24^, ^25^ To investigate whether antiviral treatment with RM‐5061 + OST affected the recruitment of influenza‐specific CD8^+^ T‐cell responses, we investigated the antigen‐specific immunodominant D^b^NP_366_^+^CD8^+^ T‐ and D^b^PA_224_^+^CD8^+^ T‐cell responses^26^ following influenza virus infection in the presence/absence of antiviral therapies (Figure 5A). We first examined the D^b^NP_366_^+^CD8^+^ T‐ and D^b^PA_224_^+^CD8^+^ T‐cell responses in the draining lymph nodes (mLN). Although there was a trend towards lower D^b^NP_366_^+^CD8^+^ T‐ and D^b^PA_224_^+^CD8^+^ T‐cell percentage (Figure 5B) and numbers (Figure 5C) in combination‐treated mice, this difference was not significantly different to other treatment groups at d7 pi. At the site of infection, however, all the animal groups receiving antiviral therapy displayed significantly reduced numbers **(**Figure 5E) but not percentages (Figure 5D) of D^b^NP_366_^+^CD8^+^ T‐ and D^b^PA_224_^+^CD8^+^ T‐cell populations when compared to the placebo group, with reduced numbers reflecting decreased viral load, inflammation, and influx of innate cells. However, in the spleen, we did find neither reduced frequencies nor cell numbers of influenza‐specific CD8^+^ T cells in antiviral‐treated animals(Figure 5F,G). Taken together, RM‐5061 + OST treatment leads to reduced numbers of IAV‐specific CD8^+^ T cells after influenza infection at the site of infection, in accordance with reduced viral load and inflammatory milieu.

## 4 DISCUSSION

In this study, we investigated the effects of a combination treatment of RM‐5061 + OST on influenza viral replication and the subsequent effects on the influenza‐specific immune responses. We found that the combination therapy was as effective as OST monotherapy in reducing viral load, inflammation and innate immunity on d5 at the site of infection, and consequently, antigen‐specific CD8^+^ T‐cell responses. RM‐5061 + OST and OST‐treated animals had an overall reduction in the amount of cytokines present in the lungs with RM‐5061 + OST‐treated animals having significantly reduced levels of RANTES. We also found a reduction in the numbers of influenza‐specific CD8^+^ T‐cell responses in all treatment groups. To our knowledge, this is the first time these two antiviral compounds have been tested in combination in vivo.

RM‐5061 is a second‐generation thiazolide that targets host‐specific factors inhibiting hemagglutinin maturation via the endoplasmic reticulum protein ERp57.^27^ First‐generation thiazolides, such as nitazoxanide, were licensed to treat parasitic infections and more recently repurposed for the treatment of viral respiratory infections.^14^, ^27^ Second‐generation thiazolides were developed for better systemic absorption to increase antiviral activity,^27^ therefore, initial studies with the second‐generation thiazolide RM‐5061 showed up to sevenfold higher blood concentration and improved bioavailability in rats, compared to its progenitor compounds.^17^ Toxicity studies have been completed in dogs and rats where up to 75 mg/kg/d or 25 mg/kg/d once daily for 28 consecutive days with no adverse effects identified.^27^ Similarly, in this study, we found that the administration of 3 mg/kg/d in mice was safely tolerated in these animals.

Combination therapy is often considered as an approach to enhance virologic activity and reduce the incidence of resistance. In this study, we used a neuraminidase inhibitor, OST and a host targeting antiviral RM‐5061. When compared to OST monotherapy, the addition of RM‐5061 as a combination did not significantly enhance responses. Similarly, the cellular innate immune responses between the two treatment groups were comparable. This could most likely be a result of the maximal effects of OST have already been reached and, as observed in RM‐5061 monotherapy group, the effects of this antiviral are subtle and might be overwhelmed by OST. In future studies, it will be important to further understand the effects of a combination therapy in a dose‐sparing regimen as well as its effect on the emergence of OST‐resistant variant viruses. Dose‐sparing experiments with sup‐optimal doses of OST would be important in deciphering the effects of RM‐5061 in combination therapy. Furthermore, in vivo studies have shown that the active ingredient of RM‐5061, Tizoxanide and OST share an additive relationship and as highlighted in our ferret experiments combination therapy reduced the likelihood of infection and therefore direct infection of animals whereby large viral titres are used to infect the animals could also be hindering our ability to see accelerated viral clearance. Potentially, models which investigate the reduction of viral transmission may be better suited to determine the efficacy of these drug compounds. The avoidance of selecting new antiviral resistant strains by combination therapy, or potential effects of combination therapy on OST‐resistant strains (eg H275Y neuraminidase mutation), would be an interesting topic of future studies, which was not addressed by the set‐up of our current study.

Interestingly, we did not find an overall reduction in the amount of cytokines present in the lungs of treated animals but a significant reduction was observed in the levels of RANTES in RM‐5061 + OST at d5 and d7 p.i.. RANTES is a key innate cytokine produced by infected epithelial cells, T cells and other immune cells^28^ and results in the recruitment and activation of T cells, granulocytes and macrophages the lung.

In our study, the numbers of influenza‐specific CD8^+^ T cells in mice treated with antivirals were significantly lower than those in placebo‐treated animals. It has been previously reported that OST treatment decreases the magnitude of the influenza‐specific immune response generated during the acute phase of viral infection but that these cells were capable of mounting a robust recall response upon secondary infection with a heterologous strain of the influenza virus.^29^

Overall, our study provides evidence that a combination therapy with host targeting compound, RM‐5061 and neuraminidase inhibitor, OST, was safe in mice but did not lead to additional reduced inflammatory responses to subsequent viral infection over OST monotherapy. Likely due diminished inflammatory pulmonary environment in OST and RM‐5061 combination treatment groups, influenza‐specific effector CD8^+^ T‐cell responses were also decreased in these animals on d7 p.i.. Combination therapy remains a feasible treatment option to reduce the morbidity and mortality associated with influenza virus infections in severely ill patients. However, future work needs to be conducted to examine the subsequent effects of antiviral treatment on the emergence of drug‐resistant mutant viruses.

## CONFLICT OF INTERESTS

ACH is an employee of Hoffmann La‐Roche. Romark Laboratories provided funds for research.

## AUTHOR CONTRIBUTION

**Simone N**ü**ssing:** Formal analysis (equal); Investigation (equal); Methodology (equal); Validation (equal); Writing‐original draft (equal); Writing‐review & editing (equal). **Edin Mifsud:** Data curation (equal); Investigation (equal); Methodology (equal); Validation (equal); Writing‐original draft (equal); Writing‐review & editing (equal). **Luca Hensen:** Formal analysis (supporting); Investigation (equal); Methodology (equal); Writing‐review & editing (supporting). **Marios Koutsakos:** Formal analysis (supporting); Investigation (supporting); Methodology (supporting); Writing‐review & editing (supporting). **Zhongfang Wang:** Formal analysis (supporting); Investigation (supporting); Methodology (supporting). **Lukasz Kedzierski:** Formal analysis (supporting); Investigation (supporting); Methodology (supporting); Writing‐review & editing (supporting). **Francesca Mercuri:** Conceptualization (supporting); Writing‐review & editing (supporting). **Jean‐Francois Rossignol:** Conceptualization (lead); Funding acquisition (supporting); Writing‐review & editing (supporting). **Aeron C. Hurt:** Conceptualization (lead); Funding acquisition (lead); Project administration (lead); Writing‐original draft (lead); Writing‐review & editing (lead). **Katherine Kedzierska:** Conceptualization (lead); Funding acquisition (lead); Investigation (lead); Resources (lead); Supervision (lead); Writing‐original draft (lead); Writing‐review & editing (lead).

### Peer Review

The peer review history for this article is available at https://publons.com/publon/10.1111/irv.12776.

## References

[1] Iuliano AD , Roguski KM , Chang HH , et al. Estimates of global seasonal influenza‐associated respiratory mortality: a modelling study. Lancet. 2018;391:1285‐1300. 29248255 10.1016/S0140-6736(17)33293-2 PMC5935243

[2] Krammer F , Smith GJD , Fouchier RAM , et al. Influenza. Nat Rev Dis Primers. 2018;4:3. 29955068 10.1038/s41572-018-0002-y PMC7097467

[3] Hay AJ , Wolstenholme AJ , Skehel JJ , Smith MH . The molecular basis of the specific anti‐influenza action of amantadine. EMBO J. 1985;4:3021‐3024. 4065098 10.1002/j.1460-2075.1985.tb04038.x PMC554613

[4] Moscona A . Neuraminidase Inhibitors for Influenza. N Engl J Med. 2005;353:1363‐1373. 16192481 10.1056/NEJMra050740

[5] Uehara T , Shionogi and Co . L S‐033188, a small molecule inhibitor of cap‐dependent endonuclease of influenza A and B Virus, leads to rapid and profound viral load reduction. In: Options for the Control of Influenza IX (ed^(eds).

[6] Oh DY , Hurt AC . A review of the antiviral susceptibility of human and avian influenza viruses over the last decade. Scientifica (Cairo). 2014;2014:1–10. 10.1155/2014/430629 PMC3995103 24800107

[7] Lackenby A , Besselaar TG , Daniels RS , et al. Global update on the susceptibility of human influenza viruses to neuraminidase inhibitors and status of novel antivirals, 2016–2017. Antiviral Res. 2018;157:38‐46. 29981793 10.1016/j.antiviral.2018.07.001 PMC6094047

[8] Takashita E , Meijer A , Lackenby A , et al. Global update on the susceptibility of human influenza viruses to neuraminidase inhibitors, 2013–2014. Antiviral Res. 2015;117:27‐38. 25721488 10.1016/j.antiviral.2015.02.003 PMC9036627

[9] Govorkova EA , Fang H‐B , Tan M , Webster RG . Neuraminidase inhibitor‐rimantadine combinations exert additive and synergistic anti‐influenza virus effects in MDCK cells. Antimicrob Agents Chemother. 2004;48:4855‐4863. 15561867 10.1128/AAC.48.12.4855-4863.2004 PMC529183

[10] Galabov AS , Simeonova L , Gegova G . Rimantadine and oseltamivir demonstrate synergistic combination effect in an experimental infection with type a (H3N2) influenza virus in mice. Antiviral Chem Chemother. 2006;17:251‐258. 10.1177/095632020601700502 17176629

[11] Fox LM , Saravolatz LD . Nitazoxanide: a new thiazolide antiparasitic agent. Clin Infect Dis. 2005;40:1173‐1180. 15791519 10.1086/428839

[12] Rossignol JF , Ayers Marc S , Ayoub A . Treatment of Diarrhea Caused by *Giardia intestinalis* and *Entamoeba histolytica* or *E. dispar*: A randomized, double‐blind, placebo‐controlled study of nitazoxanide. J Infect Dis. 2001;184:381‐384. 11443569 10.1086/322038

[13] Amadi B , Mwiya M , Musuku J , et al. Effect of nitazoxanide on morbidity and mortality in Zambian children with cryptosporidiosis: a randomised controlled trial. Lancet. 2002;360:1375‐1380. 12423984 10.1016/S0140-6736(02)11401-2

[14] Tilmanis D , van Baalen C , Oh DY , Rossignol J‐F , Hurt AC . The susceptibility of circulating human influenza viruses to tizoxanide, the active metabolite of nitazoxanide. Antiviral Res. 2017;147:142‐148. 28986103 10.1016/j.antiviral.2017.10.002

[15] Amireddy N , Puttapaka SN , Vinnakota RL , Ravuri HG , Thonda S , Kalivendi SV . The unintended mitochondrial uncoupling effects of the FDA‐approved anti‐helminth drug nitazoxanide mitigates experimental parkinsonism in mice. J Biol Chem. 2017;292:15731‐15743. 28798236 10.1074/jbc.M117.791863 PMC5612106

[16] Rossignol JF , La Frazia S , Chiappa L , Ciucci A , Santoro MG . Thiazolides, a new class of anti‐influenza molecules targeting viral hemagglutinin at the post‐translational level. J Biol Chem. 2009;284:29798‐29808. 19638339 10.1074/jbc.M109.029470 PMC2785610

[17] Stachulski AV , Swift K , Cooper M , et al. Synthesis and pre‐clinical studies of new amino‐acid ester thiazolide prodrugs. Eur J Med Chem. 2017;126:154‐159. 27750149 10.1016/j.ejmech.2016.09.080 PMC7125651

[18] Wang Z , Zhang A , Wan Y , et al. Early hypercytokinemia is associated with interferon‐induced transmembrane protein‐3 dysfunction and predictive of fatal H7N9 infection. Proc Natl Acad Sci. 2014;111:769. 24367104 10.1073/pnas.1321748111 PMC3896201

[19] Wang Z , Wan Y , Qiu C , et al. Recovery from severe H7N9 disease is associated with diverse response mechanisms dominated by CD8+ T cells. Nat Commun. 2015;6:6833. 25967273 10.1038/ncomms7833 PMC4479016

[20] Nüssing S , Sant S , Koutsakos M , Subbarao K , Nguyen THO , Kedzierska K . Innate and adaptive T cells in influenza disease. Front Med. 2018;12:34‐47. 29352371 10.1007/s11684-017-0606-8

[21] Kedzierska K , Valkenburg S , Doherty P , Davenport M , Venturi V . Use it or lose it: establishment and persistence of T cell memory. Front Immunol. 2012;3:357. 23230439 10.3389/fimmu.2012.00357 PMC3515894

[22] Wang Z , Zhu L , Nguyen THO , et al. Clonally diverse CD38+HLA‐DR+CD8+ T cells persist during fatal H7N9 disease. Nat Commun. 2018;9:824. 29483513 10.1038/s41467-018-03243-7 PMC5827521

[23] Valkenburg SA , Gras S , Guillonneau C , et al. Protective efficacy of cross‐reactive CD8+ T cells recognising mutant viral epitopes depends on peptide‐MHC‐I structural interactions and t cell activation threshold. PLoS Pathog. 2010;6:e1001039. 20711359 10.1371/journal.ppat.1001039 PMC2920842

[24] Koutsakos M , Illing PT , Nguyen THO , et al. Human CD8+ T cell cross‐reactivity across influenza A, B and C viruses. Nat Immunol. 2019;20(5):613‐625. 30778243 10.1038/s41590-019-0320-6

[25] Auladell M , Jia X , Hensen L , et al. Recalling the future: immunological memory toward unpredictable influenza viruses. Front Immunol. 2019;10:1400. 31312199 10.3389/fimmu.2019.01400 PMC6614380

[26] Kedzierska K , Venturi V , Field K , Davenport MP , Turner SJ , Doherty PC . Early establishment of diverse T cell receptor profiles for influenza‐specific CD8+ CD62Lhi memory T cells. Proc Natl Acad Sci. 2006;103:9184. 16754852 10.1073/pnas.0603289103 PMC1482587

[27] Rossignol JF , La Frazia S , Chiappa L , Ciucci A , Santoro MG . Thiazolides, a new class of anti‐influenza molecules targeting viral hemagglutinin at the post‐translational level. J Biol Chem. 2009;284:29798‐29808. 19638339 10.1074/jbc.M109.029470 PMC2785610

[28] Matsukura S , Kokubu F , Noda H , Tokunaga H , Adachi M . Expression of IL‐6, IL‐8, and RANTES on human bronchial epithelial cells, NCI‐H292, induced by influenza virus A. J Allergy Clin Immunol. 1996;98:1080‐1087. 8977509 10.1016/s0091-6749(96)80195-3

[29] Bird NL , Olson MR , Hurt AC , et al. Oseltamivir prophylaxis reduces inflammation and facilitates establishment of cross‐strain protective T cell memory to influenza viruses. PLoS One. 2015;10:e0129768. 26086392 10.1371/journal.pone.0129768 PMC4473273

